# The Potential for Outdoor Nature-Based Interventions in the Treatment and Prevention of Depression

**DOI:** 10.3389/fpsyg.2022.740210

**Published:** 2022-03-23

**Authors:** Matthew Owens, Hannah L. I. Bunce

**Affiliations:** ^1^Department of Psychology, The Mood Disorders Centre, University of Exeter, Exeter, United Kingdom; ^2^CEDAR, University of Exeter and Somerset Foundation Trust NHS, Taunton, United Kingdom

**Keywords:** nature-based, depression, prevention, intervention, mechanisms, personalised approach, children, outdoors

## Abstract

There is growing interest in nature-based interventions (NBI) to improve human health and wellbeing. An important nascent area is exploring the potential of outdoor therapies to treat and prevent common mental health problems like depression. In this conceptual analysis on the nature–depression nexus, we distil some of the main issues for consideration when NBIs for depression are being developed. We argue that understanding the mechanisms, or ‘active ingredients’ in NBIs is crucial to understand what works and for whom. Successfully identifying modifiable mediating intervention targets will pave the way for interventions with increased efficacy. We highlight a non-exhaustive list of five clinically relevant putative, candidate mechanisms which may underly the beneficial effects of NBIs on depression: stress, rumination, mindfulness, sleep and exercise. We also make the case that when developing NBIs it is important to not neglect young people, explore personalised approaches and focus on both treatment and prevention approaches. To achieve these aims methodologically rigorous programmes of clinical research are needed that include well-powered and controlled experimental designs including randomised controlled trials, qualitative research, longitudinal studies and large prospective cohorts.

## Introduction

Developing nature-based interventions (NBI) in both the treatment and prevention of common mental health problems, such as depression, is a nascent field which has potential to reduce the large burden associated with poor wellbeing and psychopathology (Frumkin et al., 2017; Bratman et al., 2019). Incorporating natural environments into clinical practice has hitherto been an overlooked area but evidence is now gathering to suggest that NBIs could be developed in clinical psychology to treat and prevent clinical depression. The mechanisms via which such interventions may deliver efficacy in mitigating deleterious effects of depression are as yet unclear, however. Nevertheless, there are a number of candidate mechanisms, that should be further explored and tested, which are implicated in both exposure to nature and the pathogenesis of depression. There is scope to further develop NBIs for clinical depression and to this end, informed by a narrative review of the extant literature, a conceptual analysis of both the nature of depression and its relationship with nature will be informative. Here, we briefly review the evidence for the beneficial effects of nature on health, wellbeing and depression and pay particular attention to putative mechanisms that may explain the link between contact with nature and depression. The search for mediating factors is vital in clinical psychology because if modifiable ‘core components’ or ‘active ingredients’ can be identified, there is then potential to harness this knowledge in the service of designing more effective interventions, whether these are prevention or treatment models, or blends thereof. In addition, we highlight key conceptual and methodological issues to be considered when designing treatment and prevention research that is situated in the nexus between nature and depression.

Circumstantial evidence for the connection between nature and depressive disorders can be found in a range of sources including a meta-analysis showing that the prevalence of mood disorders is some 39% higher in urban versus rural areas (Peen et al., 2010). However, the results from subsequent reviews have been ambivalent, some finding no association between urban/rural setting and depression (Breslau et al., 2014) others reporting more depression in urban areas but only for developed versus developing nations (Purtle et al., 2019). More recently, a review found higher common mental health disorder prevalence including depression but particularly for anxiety in more urbanised countries (van der Wal et al., 2021). A long-standing, guiding theoretical framework for understanding the connection between nature and human health and wellbeing comes from E.O. Wilson’s seminal functional-evolutionary *biophilia* hypothesis (literally a love of life), or more formally, an, ‘innate tendency to focus on life and lifelike processes’ (Wilson, 1984). In addition, recognising both positive and negative aspects of biophilia (i.e., *biophobia*), a complementary yet nuanced conceptualisation is that of *protoaxis*, or the tendency for organisms to react to each other in distinct ways (Sagan and Margulis, 1993). Inherent to these perspectives is a fundamental assumption that affinity with nature, however defined, is adaptive and genetic in origin. Precisely which constituent parts of natural environments are most important in conferring benefit to individuals is unclear and the subject of much research. For example, in a review of the evidence, a research agenda was set out that moves beyond visual experience to include the remaining four senses (sound, smell, taste and touch) as well as non-sensory pathways such as exposure to stress-reducing phytoncides (Franco et al., 2017).

A narrative review of the current empirical evidence suggests that contact with nature can bestow a plethora of beneficial effects on both physical and mental health. Although the precise processes are not yet clear, it is thought that contact with nature may confer positive effects via stress reduction (Ulrich et al., 1991; Berto, 2014), restoration of cognitive and emotional functioning following stress and mental fatigue (Kaplan, 1995), perhaps mediated by the latent psycho-evolutionary affinity with natural environments, otherwise known as nature connection (Capaldi et al., 2014). More recently, evolutionary models that take affect regulation more fully into account have been posited (Richardson et al., 2016). It is important to recognise that what constitutes ‘natural environments’ is complex and multifaceted. For example, a number of factors should be considered including total area, composition, spatial configuration, tree canopy density, species composition and biodiversity (Bratman et al., 2019). Although beyond the scope of the present narrative review, determining which features of nature are most beneficial to mental health is a crucial current research priority (Bratman et al., 2019).

The modern era has seen more than half of the global population move to urbanised areas over the last century and the rapid rise over technology has meant a sizeable reduction in exposure to natural environments for most people (Colding et al., 2020). We now spend as much as 90% of our time indoors (Frumkin et al., 2017) and children are detached from nature to the extent that they know far more about Pokémon than natural species (Balmford, 2002). Not only is urbanisation set to increase in years to come, but parents are also limiting children’s access to nature to protect them from harms, real or imagined (Veitch et al., 2010). One concern is that we continue to become more disconnected from nature, when natural environments may be inherently beneficial for the health and wellbeing of humans. It should be pointed out here that a potential mitigating strategy against urbanisation is to attempt to bring nature indoors. It has been shown for example that bringing plants into office spaces or ensuring office workers have an outdoor green view is associated with less stress and more happiness (Hall and Knuth, 2019).

The idea that contact with nature is positive for human health is not new. One of the earliest recorded pioneers was Cyrus the Great in the sixth century BC, who built lush, enclosed gardens to foster relaxation and calm away from the busy city (Rostami et al., 2016). Hippocrates stressed the importance of a scenic environment for improving one’s health (Burford, 1969) and gardens in 13th-century monasteries were viewed as sources not only to heal the sick and preserve health but also to, ‘improve those fatigued by their spiritual studies’ (Montford, 2004). Interestingly, in the 16th Century Swiss-German physician Paracelsus went further, suggesting that, ‘the art of healing comes from nature, not from the physician’ (Hansen et al., 2017). More recently, there has been a resurgence in interest in how contact with nature may facilitate health and wellbeing.

Perhaps one of the most striking potential features of contact with nature, and sharing some commonality with other ‘lifestyle’ factors, are the seemingly pleiotropic beneficial health effects it may have on general health (Cox et al., 2017), including but not limited to improved birth outcomes (Dzhambov et al., 2014; Twohig-Bennett and Jones, 2018), asthma and allergies (Cavaleiro Rufo et al., 2021), improved immune functioning (Li et al., 2008; Li and Kawada, 2011; Hall and Knuth, 2019), diabetes (Brown et al., 2016; Thiering et al., 2016; Tsai et al., 2021), lowering blood pressure (Shanahan et al., 2016), reduction in pain perception acutely (Lechtzin et al., 2010) and chronically (Wells et al., 2019), improving postoperative recovery (Park and Mattson, 2009) and reduced mortality (James et al., 2016; Crouse et al., 2017). Furthermore, there is evidence to suggest beneficial effects on a number of possible mechanisms of action implicated in the aetiology and pathophysiology of depression, including reducing stress (Yao et al., 2021), inflammation (Stier-Jarmer et al., 2021) and obesity (Schalkwijk et al., 2018), improving wellbeing (Martin et al., 2020; Pritchard et al., 2020; Pirchio et al., 2021) and improved sleep (Shin et al., 2020). Coupled with the emerging literature that provides promising evidence suggesting a benefit for depression (Hossain et al., 2020; Olafsdottir et al., 2020; Williams et al., 2020; Antonelli et al., 2021; Rosa et al., 2021; Stier-Jarmer et al., 2021), NBIs appear to be a viable strategy to pursue in mitigating the personal, social and societal burden of depression. We first outline the problem of depression and identify important issues that should inform the development of future interventions. We then discuss the emerging evidence on NBIs, some of the potential mechanisms of action and methodological and conceptual considerations.

## Depression: A Complex Problem

Mental health problems are highly prevalent, and depression is one of the most common (Wittchen et al., 2011; Kessler and Bromet, 2013) with estimated annual costs made in 2016 standing at a staggering $1 trillion (Chisholm et al., 2016). Indeed, clinical depression is currently the leading global health problem (World Health Organization, 2017) and associated with a litany of adverse sequelae including poor education, relationship disruption, unstable employment and an increased risk of mortality through somatic illness and suicide (Kessler, 2012). The problem is exacerbated because more than half of individuals develop recurrent depression, spending a fifth of their life with a depressive disorder (Cuijpers et al., 2012).

Depression is a highly complex and heterogeneous disorder not fully understood but has genetic and environmental influences (Sullivan et al., 2000) with its roots found in the early years (Dunn et al., 2011). Furthermore, depression in youth may be rising (Bor et al., 2014; Patalay and Gage, 2019) and young people are particularly vulnerable. Despite approximately three-quarters of mental health disorders appearing before the age of 24 (Kessler et al., 2005a), treatment is not typically initiated until some years later, if at all (McGorry et al., 2011), which has prompted the piloting of dedicated youth mental health services (Maxwell et al., 2019). In addition, mental health problems including depression have increased recently, in both children and adults, as a result of the response to the COVID-19 pandemic (Pierce et al., 2020; Townsend, 2020; Niedzwiedz et al., 2021).

While many suffering with depression do receive benefit from treatment approaches, the proportion of patients in remission following psychological or pharmacological treatment is typically less than 50% (Kolovos et al., 2017; Van Bronswijk et al., 2019). It is also perhaps somewhat disappointing to note that the heightened investment in treatment initiatives in recent years has not corresponded to a reduction in depression prevalence or the burden associated with it (Ormel et al., 2020). Unfortunately, this means that most people in need of treatment for mental health problems do not actually receive any (Kessler et al., 2005b). With a realisation that relying on a treatment only is unlikely to be enough to help everyone in need, adding in depression *prevention* interventions has now become a global priority (Cuijpers et al., 2012; Ebert and Cuijpers, 2018). It is therefore important to consider both treatment and prevention approaches when designing NBIs for depression.

A further fundamental factor compounding this problem is the so-called *heterogeneity* problem in depression. As David Goldberg has put it, ‘At present major depression has become a monolith…it may be politically important to utter such simplifications to doctors in general medical setting, but it is a convenient fiction’ (Goldberg, 2011). Heterogeneity in depression can also mean heterogeneity of the trajectory through intervention and outcome. Heterogeneity is the *sine qua non* of the *personalised* approach to healthcare (Cuijpers and Christensen, 2017), otherwise known as precision healthcare, tailored healthcare, *n*-of-one or treatment matching. This approach recognises the importance of individual differences and asks the question, ‘what works for whom?’ (Roth and Fonagy, 2006). If adopted by clinicians and scientists, the personalised approach could significantly boost the impact and uptake of evidence-based interventions whether they are treatments (Cuijpers and Christensen, 2017) or prevention approaches (August and Gewirtz, 2019).

A second recent development and approach of the problem of lower than optimal efficacy rates in depression interventions (traditionally focussed on cognitive behavioural approaches) has been to place more emphasis on lifestyle factors, which encompasses NBIs. This growing area, lifestyle psychiatry, or *lifestyle psychology* aims to investigate alternative approaches to intervention. Lifestyle psychology (Lopresti et al., 2013; Firth et al., 2020) traditionally includes three prominent areas of diet (Jacka, 2017; Ventriglio et al., 2020), sleep (Cheng et al., 2020; Reynolds et al., 2020) and exercise (Kandola et al., 2019; Wegner et al., 2020), all of which are active fields in their own right. By way of a parallel example, in the field of nutritional psychology, it is thought that following a healthy diet (e.g., a Mediterranean style diet) is beneficial for mental health and wellbeing (Marx et al., 2017). The potential for nutritional strategies as either treatment or prevention is currently being explored (Jacka et al., 2017; Bot et al., 2019), although the underlying mechanisms are yet to be determined (Owens et al., 2020, 2021). More recently in lifestyle psychology, there have been calls to increase efforts exploring more novel interventions, including contact with nature (Piotrowski et al., 2021). It is therefore important to understand the current landscape in terms of NBIs and highlight the key considerations as they apply to the treatment and prevention of depression.

In sum, depression is a complex, heterogeneous problem that typically emerges in youth. This inherent heterogeneity means that it is imperative to understand which interventions work best for whom and so personalised approaches should also be explored. Current treatments are limited in their efficacy and in addition, limited resources means that collectively we are unlikely to be able to treat all of those in need, if for no other reason than the constant influx of new cases (Moreno-Peral et al., 2020). For these reasons, novel interventions including prevention strategies are urgently required and NBIs for depression may complement existing approaches.

### Nature-Based Interventions

NBIs may include approaches that have the potential to improve functioning in typical, non-clinical samples (Richardson et al., 2020), including young people (Mygind et al., 2019; Zhang et al., 2020), as well as therapeutic approaches aimed to treat clinical depression. For example, in a small sample of 20 adults diagnosed with clinical depression, Berman et al. (2012) found that after inducing negative rumination in all participants, improved positive affect was subsequently reported in those randomised to a walk in nature versus an urban walk. Other approaches speak to both treatment and prevention. For instance, in a Japanese uncontrolled proof-of-principle study, Furuyashiki et al. (2019) showed that after a day-long forest bathing intervention, mental distress symptoms were reduced both for those with high and low levels of mental distress, indicating promise for both prevention and treatment.

Reviews of randomised and non-randomised studies have suggested that NBIs can decrease depressive symptoms in both clinical and non-clinical samples (Van Tulleken et al., 2018; Hossain et al., 2020; Roberts et al., 2020; Wolf et al., 2020; Rosa et al., 2021). A total of 14 systematic reviews and meta-analyses on outdoor therapeutic approaches were identified in a recent review of children and adults (Harper et al., 2021). The majority of these approaches (nature-based therapy, wilderness therapy, horticulture therapy, adventure therapy, nature-based mindfulness and forest bathing) included some evidence of positive mental health and wellbeing outcomes. A major conclusion of this umbrella review, however, highlighted that ‘clear and comprehensive descriptions of theory, program structure, and activity details with causal links to outcomes were mostly absent’ (Harper et al., 2021). Particularly problematic was the finding that sufficient clarity on the potential mediators and mechanisms of change for outcomes in all studies was lacking. As the authors rightly point out, outdoor settings are complex social and environmental settings with an almost infinite set of potential explanatory variables. For this reason, it is imperative to design research studies that are robust enough to test these key mediation hypotheses.

Understanding the underlying mechanisms in intervention research is complex but necessary for several reasons and using mediation analyses to achieve this is recommended in treatment and prevention research (Kraemer et al., 2002; O’Rourke and MacKinnon, 2018). Mediation analyses facilitate the testing of known as well as hypothesised mechanisms (e.g., attention restoration, nature connection), provide an opportunity to perform ‘manipulation checks’ to test whether an intervention has changed the hypothesised factor that it was designed to (MacKinnon, 1994) and perhaps most importantly to inform the development of theory. Identifying mediators in NBIs is of paramount importance in that this can help identify key processes to target and enhance the potency of intervention effects on depression outcomes. It should also be noted that to help progress theory, hypothesised intermediate processes should be investigated in every intervention study, even in the absence of a treatment effect for both statistical and conceptual reasons (O’Rourke and MacKinnon, 2018).

### Clinically Relevant Potential Mechanisms

A number of known and emerging factors exist for the development and maintenance of clinical depression. To successfully identify variables that mediate the link between exposure to nature and depression requires linking the candidate to *both* nature and depression. Such mechanisms could be important modifiable targets in NBIs which can be tested for in rigorous, controlled studies. Several candidates exist in the literature but five that are highly clinically relevant to depression are outlined below.

#### Stress

Experiencing stressful life events is a well-known risk factor for depression (Kendler et al., 1999; Hammen, 2006), which makes stress reduction/recovery a plausible and likely mechanism of change in this context. Supporting this hypothesis, there is gathering evidence that NBIs do have the ability to reduce both psychological and physiological stress (Corazon et al., 2019; Yao et al., 2021). Furthermore, the literature on forest bathing also suggests reliable reductions in cortisol using this approach (Antonelli et al., 2019), which is potentially important in depression because hypersecretion of cortisol in depressed patients (Firth et al., 2020) is one of the most reliable findings in all of psychiatry (Stetler and Miller, 2011). Elevated cortisol in combination with the presence of depressive symptoms may also predict onset of disorder in young people (Owens et al., 2014). Therefore psychological, or perceived stress as well as this specific biomarker should continue to be measured in NBI depression research and their mediating role carefully assessed.

#### Rumination

Depressive rumination, defined as the tendency to repetitively analyse the causes, meanings and consequences of problems and symptoms of depression (Watkins, 2008), is a well-established and important mechanism in depression which has been robustly implicated in its onset and maintenance (Watkins and Roberts, 2020). Rumination can be conceptualised as one form of avoidance behaviour that can result in, for example social withdrawal, retreating to bed and drinking alcohol or excessive eating. This may reduce distress in the short-term but contribute to the maintenance of an emotional disorder in the long-term (Moulds et al., 2007; Clancy et al., 2016).

Interestingly, several NBI studies but not all (Golding et al., 2018) have already reported reductions in rumination in different contexts (Wells et al., 2019; Bratman et al., 2021; McEwan et al., 2021). McEwan (2021) for example, randomly assigned a sample of university staff and students to one of forest bathing, compassionate mind training or both in their pilot study. Among the positive changes found for *all groups*, rumination on problems was reduced, indicating that forest bathing alone was equally as effective as a psychological therapy for depression in reducing repetitive negative thinking. It has also been shown that rumination can be reduced through walking in nature versus urban settings and this decrease may be mediated by reductions in the subgenual prefrontal cortex, an area implicated in depressive rumination (Bratman et al., 2015). More recently, in a cross-sectional mediation analysis, Bratman et al. (2021) provided evidence to suggest that the association between time spent in nature and positive and negative affect was partially explained by levels of rumination. Taken together the current evidence suggests that reductions in rumination may be a key mechanism responsible for the NBI-associated improvements seen in depressive symptoms and may be important for both prevention and treatment.

#### Mindfulness

Mindfulness is defined as the awareness that arises through ‘paying attention in a particular way: on purpose, in the present moment, and non-judgementally’ (Kabat-Zinn, 1994). Meta-analyses have shown that mindfulness traits and nature connectedness are positively associated, which opens up the possibility of building NBIs that also have the aim of increasing levels of mindfulness to treat or prevent depression (Schutte and Malouff, 2018). The authors of this meta-analysis postulate that the association between mindfulness and nature connectedness is explained by non-judgemental awareness, which may facilitate engagement with nature. This suggests a reciprocal relationship between mindfulness and nature connection, a hypothesis that can be tested in future research. It should also be noted that mindfulness is an inherent part of the practice of shinrin-yoku, or forest bathing (Kotera et al., 2022). It is important to tease out which combination of components in NBIs, such as forest bathing (e.g., walking, sensory experience, mindful awareness, improved attentional control and reduced rumination), are optimal in future depression research.

Mindfulness-based interventions (MBI) is an umbrella term to describe a range of interventions including for example, Mindfulness-Based Cognitive Therapy (MBCT) and Mindfulness-Based Stress Reduction (MBSR). MBIs are now ubiquitous within clinical settings and have demonstrated efficacy in treating depression (Khoury et al., 2013). They have been shown to be non-inferior to evidence-based treatments for depression including antidepressant medication (Kuyken et al., 2015) and cognitive behavioural therapy (CBT) (Goldberg et al., 2018). One psycho-social MBI for depression recommended by the National Institute for Clinical Excellence (NICE, 2018) is MBCT, an eight-week group program for the treatment and relapse prevention of depression. A meta-analysis assessing potential mediators of the beneficial effect of MBCT and MBSR on mental health and wellbeing found strong evidence for several clinically relevant factors including cognitive and emotional reactivity, mindfulness, rumination and worry (Gu et al., 2015). Another potentially relevant mechanism of change, related to mindfulness is self-compassion (Evans et al., 2018), which may be applicable to future nature research.

It is important to note here that research suggests that it is specifically the *awareness* and not the *acceptance* component of mindfulness that is more strongly associated with nature connectedness (Howell et al., 2011). Non-judgemental awareness is a form of attention with neural correlates in both focusing attention and attentional control (Wenk-Sormaz, 2005; Moore and Malinowski, 2009; Teper and Inzlicht, 2013), which is consistent with the central tenet of attention restoration theory (ART) (Kaplan, 1995). ART suggests directed attention is a limited resource that requires restoration and contact with nature may have a role in facilitating this process, by allowing indirect attention to take precedence, thereby restoring directed attention. Evidence from systematic reviews on ART, however, has been equivocal. Some evidence suggests improvements after contact with nature for working memory, cognitive flexibility and attentional control but not for other measures of attention (Ohly et al., 2016; Stevenson et al., 2018). For example, Ohly and colleagues found support for the positive effect of nature on a range of measures of attention in only three out of 13 meta-analyses. Future research should continue to explore which executive functions are implicated after exposure to nature. For example, attentional bias toward negative stimuli is thought to play a causal role in depression (Mennen et al., 2019). Similarly, training depressed patients to remove negative content from working memory may reduce physiological response to stress (Jopling et al., 2020).

There is now some suggestive evidence that mindfulness effects are enhanced in nature. In an MBSR study of six one-hour weekly sessions, non-clinical participants were randomised to one of three groups, the location of which was either indoors, a built outdoor environment or a natural outdoor environment (Choe et al., 2020). The authors completed a follow-up at 1 week and 1 month and found a larger reduction in rumination and stress in the natural outdoor environment than the other groups. Nature connectedness was increased, and positive effects of MBSR, such as decreased rumination and stress, were found to last longer in the natural outdoor group. However, although all groups improved in terms of depression, there was no significant effect of group on depression over time. These findings warrant further investigation to test these effects in a clinical population and additionally to explore limitations pertaining to potential confounder variables, such as noise levels or expansiveness of the test area. While the direction of causality of effects between nature connectedness and mindfulness is as yet unclear, exploring cognitive processes may be key to understanding underlying mechanisms. Future research should explore these possibilities in the context of depression.

#### Sleep

Sleep disturbance is ubiquitous in mental health problems (Freeman et al., 2020) and reported in the overwhelming majority of patients with depression (Lopresti, 2019) including young people (Orchard et al., 2020). Although a common perception in mental health is that sleep disturbance is solely a symptom of depression, poor sleep can in fact be both a symptom as well as a risk factor for the onset of depressive episodes. That is, there exists a complex bidirectional relationship between the two factors (Alvaro et al., 2013; Fang et al., 2019; Difrancesco et al., 2021). Encouragingly, intervention research has shown that sleep disturbance improves following treatment for depression (Reynolds et al., 2020) and depression symptoms reduce after treatment for insomnia (Gee et al., 2019; Kodsi et al., 2021). Given that sleep disturbance is a key mechanism in depression, NBIs should be carefully explored as to whether they can mitigate the deleterious effects of poor sleep on mood and wellbeing. Although to our knowledge there are no large scale NBI randomised controlled trials (RCT) specifically designed to assess the effect on sleep, a recent review on the cross-sectional and intervention studies literature reported a positive association between greenspace and good sleep, which is promising (Shin et al., 2020). For example, a small study (*n* = 13; Gladwell et al., 2016) that randomised participants to either an urban or green walk (grassland, wooded areas and a small lake) found improved night-time indices of autonomic nervous system functioning (increased heart rate variability). Clearly, larger RCTs and longitudinal studies are needed to fully test these potential mechanisms, but the existing literature is nevertheless encouraging.

#### Exercise

Exercise and physical activity are often but not always (Toseeb et al., 2014) associated with positive mental health (Tamminen et al., 2020). Several reviews have also shown that exercise interventions can be effective as treatments for managing depression as well as preventing it (Ashdown-Franks et al., 2020; Hu et al., 2020; Pascoe et al., 2020). A number of intermediate physiological and psychological processes have been proposed to mediate this effect, including reducing oxidative stress and inflammatory processes, regulating HPA axis functioning and promoting neuroprogression; self-efficacy, self-esteem, social support and emotional regulation (Lubans et al., 2016; Kandola et al., 2019; Wegner et al., 2020). Interestingly, many of these may well be shared mechanisms under certain conditions in nature exposure (Hansen et al., 2017) but research has yet to fully integrate these factors with different elements of NBIs.

Given that the landscape in which NBIs operate is multifaceted, it is important to tease out which factors are operating in a given intervention. For example, walking in nature clearly involves (as a minimum) the two components of physical activity and exposure to a natural environment. The extent to which either, both, or their combination is most predictive of positive mental health outcomes is a good example of how unpicking the complexities involved is important. In a non-clinical sample of university students, an RCT carried out by Olafsdottir et al. (2020) tested the relative restorative effects of walking in nature, watching nature virtually, versus physical activity alone. The results of this study showed that all groups produced reductions in negative affect immediately following the intervention yet walking in nature improved mood more than watching nature scenes or physical activity alone, suggesting a multiplicative effect on mood between physical activity and nature exposure. Interestingly, the majority of the significant effects in this study were not maintained at follow-up, underscoring the necessity of designing RCTs with long follow-up periods. In addition, more longitudinal studies are warranted. Similarly, in a meta-analysis (*k* = 13 studies, based in Korea exclusively), it was shown that forest therapy, on average, reduced depressive symptoms more than engaging in similar activities in a hospital or non-forested urban area, or participating in an intervention focused on diet plus forest-based exercise (Rosa et al., 2021).

We do not suggest that the potential candidate mechanisms relevant to preventing and treating clinical depression discussed here represent an exhaustive list but we believe that they currently have a strong enough evidence base to test in the first instance. We recognise that there are other factors that are likely to be important in influencing outcomes, including, for example, self-esteem and self-efficacy (Fiorilli et al., 2019; Mygind et al., 2019), inflammation (Lamers et al., 2019; Stier-Jarmer et al., 2021), obesity (Milaneschi et al., 2019; Luo et al., 2020) and emotional regulation (Gilbert et al., 2008; Richardson et al., 2016). Furthermore, it is likely that there exist synergistic relationships between factors. That is, multiple pathways may exist. For example, contact with nature may increase mindfulness, in turn reducing inflammation; exercise may be enhanced in nature and both factors may lead to reductions in depressogenic psychological rumination. Better sleep quality could ensue and via multiple routes, stress levels may reduce. Ultimately such a holistic approach may help to reduce the burden of depression.

### Future Directions

A number of methodological and conceptual factors will be important to bear in mind for future research. We outline some of the most salient and pertinent to the forwarding of a research agenda in nature-based depression intervention. As we have discussed in this article, we agree with others (Howell et al., 2011) on the importance of investigating mediators and moderators of nature connectedness and mental health and highlight selected further areas to focus on below.

#### Study Quality, Statistical Power and Study Design

The majority of systematic reviews and meta-analyses on NBIs have highlighted methodological weaknesses in the research including overall low quality of the research (Roberts et al., 2020; Harper et al., 2021; Stier-Jarmer et al., 2021). For example, the AMSTAR quality results in the meta-review by Stier-Jarmer et al. (2021) on the preventative and therapeutic psychological and physical effects of forest-based interventions showed that study quality was only *moderate* in two, *low* in six and *critically low* in three reviews. The majority of studies were also rated as *weak* in the review of Roberts et al. (2020). In this review, several specific issues were raised as causes for concern including lack of control groups, insufficient controlling of confounding factors and the absence of follow-up data. Clearly, to have confidence in findings and to further the field, more work is needed on improving the quality of the overall research.

A fundamental issue in this area is sample size and statistical power (Roberts et al., 2020; Kotera et al., 2021; Stier-Jarmer et al., 2021). The sample sizes in the studies reviewed by Roberts et al. (2020) were generally small and very small in younger age groups. Some of the quantitative studies had larger samples (but mean *n* < 100); however, typically these did not report on statistical power. Similarly in the review on forest bathing by Kotera et al. (2021), sample sizes were small, less than 100. Small samples can be problematic given that power to detect effects may be insufficient and often becomes an issue when looking to detect assumed small effect sizes. Statistical power refers to the ability (probability) of a study to detect the effect of interest (e.g., a therapeutic effect of an outdoor therapy on depressive symptoms), if it actually exists and by convention, typically 80 or 90% power is specified. However, in psychology and neuroscience, studies tend to be underpowered (Button et al., 2013) and median power has been reported to be only 0.12 and 0.44 to detect small and medium effects, respectively (Szucs and Ioannidis, 2017).

Studies can suffer further from the lack of statistical power when looking for synergistic effects which might be expected in NBI research. For example, in a simulation study, it has been shown that if a study has 80% power to detect a main effect, there is a 29% chance of detecting an interaction between variables as large as the main effect and considerably less chance (~10%) if the interaction effect is half as large (Brookes et al., 2004). This illustrates how very underpowered RCTs can be to detect interactions and shows that they are prone to type II error. Future trials should ensure that they are fully powered to detect both main and interaction effects.

The call for more longitudinal studies has been made in many quarters (Shanahan et al., 2016; Hansen et al., 2017; Nieuwenhuijsen et al., 2017; Norwood et al., 2019; Roberts et al., 2020; Holland et al., 2021). Broadly speaking, the majority of epidemiological studies have been cross-sectional and intervention studies often include only limited follow-up timepoints. For example, in the review of intervention studies by Djernis et al. (2019), the follow-up period in the majority of studies was weeks rather than months or years. Although longer follow-ups are also desirable in depression research, they are typically longer in this area. For example, in a review of CBT trials in depressed adolescents the average follow-up period was 9.59 months (Keles and Idsoe, 2018). The results of the review showed average significant benefit for CBT immediately and at follow-up, although the latter effect was significantly attenuated. This underscores the need for longer-term follow-up when evaluating NBIs. Furthermore, it will be valuable to design more studies incorporating prospective cohorts to test the predictive value of contact with nature with mood and wellbeing and the incidence of depressive disorder. There are a number of such cohorts focusing on depression including in adults (Penninx et al., 2021) and adolescents (Lewis et al., 2016). We encourage more large, long-term cohort studies in the future to help plot out the aetiology, developmental trajectory and consequences of contact with nature.

In summary, we join others in encouraging further use of larger controlled RCTs, longitudinal studies and cohorts, qualitative analyses and advanced multivariate modelling to understand the complex and likely multiple pathways involved from nature to good mental health and wellbeing.

#### Personalised Intervention

Psychological treatments of different modality as well as pharmacological treatments for depression seem to be equally effective, on average, even though there is substantial heterogeneity in efficacy at the individual patient level (Cuijpers and Christensen, 2017). The personalised approach to both treatment and prevention rests on a fundamental assumption that the efficacy of marginally beneficial interventions can be greatly enhanced when individual differences are taken into account. The true intervention effect may be obscured by the average effect, which is often surrounded by significant heterogeneity. It is thought that maximising effectiveness is more likely when sources of individual difference are taken into account. These might include environmental experience, genetics, lifestyle, physiology, cognition, dimensions, such as connection with nature, dose, treatment preference or time of delivery (August and Gewirtz, 2019). By matching elements of interventions to individuals most likely to benefit, a therapy or prevention intervention may be more effective (Lopresti, 2019).

Cooley et al. (2020) provide a useful framework for NBIs in clinical settings. In view of a personalised approach, they suggest assessing suitability of both the client and client–practitioner dyad for working outdoors, in addition to the level of nature activity (passive/active) for treatment. This suggests how individuals might benefit from more treatment choices, embodying person-centred care (Gondek et al., 2017). Furthermore, engaging in the co-creation of treatment planning incorporates patient preference, which has been linked to favourable outcomes, better treatment adherence and satisfaction (Delevry and Le, 2019). In line with a personalised approach, offering NBIs, especially in the outdoors may assist in redressing the power imbalance typically maintained in conventional clinical settings (Johnstone et al., 2018).

A number of avenues are possible for exploring personalised approaches in depression research including stratifying on individual differences, the use of preference trials (Delevry and Le, 2019), profiling subtypes (Lamers et al., 2019) and multivariate techniques, such as latent class analysis (Connell et al., 2018; Göbel and Cohrdes, 2021). As an example in a youth sample, Young et al. (2021) categorised participants as high or low on cognitive or interpersonal risk and then randomised them to either a cognitive behavioural intervention or interpersonal psychotherapy. Those participants ‘matched’ between categorisation and randomisation arm showed a greater reduction in depressive symptoms over the course of the trial that those ‘mismatched’. Analogously, individual differences related to nature environments may also moderate response to NBIs. For example, those low on a nature connectedness trait may benefit most from an intervention. Alternatively, it might be that preference for blue versus green space determines response to an NBI offering both options.

#### Children and Young People

As previously discussed, a significant proportion of depression cases begin early in life, increasing in prevalence throughout childhood and into adolescence (Hankin, 2015). It is imperative for research not only to focus on early intervention treatments for children and young people (CYP) due to the risk of future relapse (Burcusa and Iacono, 2007) but also to develop and evaluate prevention strategies and programs. This can entail both reducing the risk of depression, such as poverty, and child abuse and increasing protective factors, such as self-esteem and stress-management skills (World Health Organization, 2004).

Research into NBIs broadly and CYP is an emerging field and all the more significant given the relationship between children and nature is in flux. Not only has children’s overall free play time reduced through the presence of more structured time, but unstructured time is more sedentary (Burdette and Whitaker, 2005) and consists of screen time and ‘videophilia’ (Pergams and Zaradic, 2006). Time spent outdoors for children and contact with nature is in decline (Edwards and Larson, 2020) yet the growing evidence for nature-derived health benefits for children, such as improvements to mental, physical and social health (Mygind et al., 2019) and promotion of resilience and recovery after traumas (Chawla, 2014) necessitates further research. Furthermore, simply living in close proximity to nature can offer benefits to children for anxiety and depression and particularly for those with low socio-economic status (Maas et al., 2009). There is a dearth of empirical investigation into the potential intervention effects for CYP with clinical depression. In the umbrella review of outdoor therapies (Harper et al., 2021) only one review was highlighted that focussed solely on children and included mental health outcomes (Mygind et al., 2019). Studies in this review included small samples and were rated as having a serious risk of bias and overall low quality. Larger rigorous studies, including RCTs, are needed to test NBIs in both samples with elevated symptoms and CYP who attract a clinical depression diagnosis.

While there is very limited evidence for depression NBIs for CYP, the circumstantial evidence suggests that a downward extension of the application of principles of contact with nature for depression from adults to children will be a reasonable approach to test. For example, analogously to the adult literature, contact with nature is accompanied by reductions in stress in children (Dettweiler et al., 2017), with children also echoing similar benefits to adults, such as escaping the pressures of everyday life (Rantala and Puhakka, 2020). Comparably, passive nature exposure for children effected positive changes in mood, attention and memory (Norwood et al., 2019).

More broadly, research from the nature-based learning (NBL) literature suggests that outdoor activities in nature promote cognitive skills and academic performance in children and that outdoor adventure programmes may improve individuals’ attitudes, beliefs and self-perceptions as well as greater social and interpersonal skills (Lovell et al., 2010). This evidence base points toward more areas for potential exploration of mediators between contact with nature and depression in children, such as cognitive factors and self-esteem. Forest schools (FS) are an example of an NBL which provide regular outdoor learning for children and there is suggestive evidence that FSs may improve the mood of children when compared with a typical school day (Roe and Aspinall, 2011). Research has shown that children participating in FS displayed increased self-confidence, improved social skills, motivation and communication (Murray and O’Brien, 2005). Again, these factors may be fruitful for further investigation of their potential role in nature connectedness and depression.

While there is evidence for positive effects of contact with nature for children, there have been some neutral and negative findings in the literature. A recent preliminary study found children showed both positive and negative emotions after attending a nature school versus a museum visit (Dopko et al., 2019). Negative emotions were hypothesised to be related to some children’s inexperience in nature and their fearfulness about seeing bears, snakes or wolves, (biophobia). The apparent disconnect of children from nature is necessary to explore further within the biophilia hypothesis. It may be that not all children necssarily benefit from or have a natural affinity for nature. For example, in a study looking at increasing physical activity through either playground sports or nature-based orienteering, Barton et al. (2015) found no additional benefit of nature-based play over and above that derived from playground-based interventions in improving physical activity nor self-esteem. Understanding more about which factors impact upon ‘what works for whom’ is important in the context of a generation for whom nature may be a novel experience, to better tailor our intervention and prevention strategies. One study utilised a qualitative methodology to explore children’s experiences retrospectively from an adult perspective to understand key factors in maintaining children’s participation in and relationships with nature and the transition into adulthood (Lovelock et al., 2016). While this may provide evidence for systemic factors, such as the role of the family and early exposure to nature, it is nevertheless important to conduct research studies with children directly, to reduce bias through self-report and recall issues and to encourage nature research with children.

#### Dose–Response

It is not yet clear what the optimal dose of nature is to promote wellbeing, let alone what the optimal elements of nature exposure are or how important individual differences are (Barton and Pretty, 2010; Holland et al., 2021). Nevertheless, these are key questions for future research to answer in order to build NBIs that are potent enough to mitigate the negative impact of depression. The question of ‘how much is enough’ is complicated when considering the range of different approaches. For example, five-minute ‘green exercise’ may be beneficial for lifting mood (Barton and Pretty, 2010) but forest bathing sessions may last 2–4 h (Antonelli et al., 2021) or be delivered in day-long sessions (Furuyashiki et al., 2019). White et al. (2019) tentatively suggested a threshold of 120 min spent in nature per week (via any summed combination of duration and frequency) and Antonelli et al. (2021), while acknowledging that as little as 15 min of forest bathing may be beneficial for wellbeing, ultimately recommend 2 h of forest bathing per week. It may be important to consider differences between specific populations and the factors being measured, as these may vary and contribute to a personalised approach. For example, in a longitudinal study with toddlers, a positive dose–response was found for hours spent outside and attention for toddlers (Ulset et al., 2017); however, the effect may be different for older children or adults.

In addition, assessing the dose–response relationship is critical in order to evaluate the causal status of the nature–depression association. As expressed in the nine viewpoints for determining causality in epidemiology (Hill, 1965), evidence of a biological gradient strengthens the case for causality. For example, after inducing stress and exposing participants to street scenes with varying levels of tree coverage (from 2 to 62%), Jiang et al. (2016) found a positive linear relationship between tree coverage and stress recovery. It has been estimated that depression could be prevented to the tune of somewhere between 5 and 27% if city residents spent between 10 and 30 min more per week visiting green spaces (Cox et al., 2017; Shanahan et al., 2019).

Dose–response relationships are also helpful in determining thresholds for beneficial amounts of nature contact. This is illustrated in the study by Cox et al. (2017) where dose–response relationships were reported for depression on several indices of contact with nature including frequency, duration and intensity of exposure to nature. Estimated incidence of depression was lower given at least one visit to green space per week, more than 5 h per week and where vegetation cover exceeded 15%. Future research should continue to measure both frequency and duration and intensity of contact with nature and further explore the exact elements that have potency or the maximal combinations. As the authors of the study note, this study was cross-sectional and therefore would benefit from further testing in a longitudinal design.

## Conclusion

There is considerable potential for nature-based treatments and prevention interventions for clinical depression and these may be helpful either as a further treatment option or as an adjunct to established therapies. Depression is a complex heterogenous mental health problem and so matching intervention to the individual is fundamental to increasing the overall efficacy of interventions. A treatment-only model in depression is not viable for resource as well as ethical reasons and so prevention in community samples as well as treatment in clinical populations should be pursued. There is currently a dearth of quality nature-based research in young people and given that depressive disorder tends to emerge at this sensitive time, there will be value in increasing the amount of research in this age group. We have highlighted some of the potential mechanisms of action in depression and recognise that the search is ongoing and should continue. Well-powered studies are required to test for the likely complex interplay between factors. Improving the overall methodological quality of intervention, qualitative, experimental and longitudinal studies will no doubt lead to discoveries that will advance what is a fundamentally important field.

## Author Contributions

MO and HB developed the conceptual analysis. MO drafted an early version of the manuscript. HB reviewed and developed the manuscript. Both authors contributed to the article and approved the submitted version.

## Conflict of Interest

The authors declare that the research was conducted in the absence of any commercial or financial relationships that could be construed as a potential conflict of interest.

## Publisher’s Note

All claims expressed in this article are solely those of the authors and do not necessarily represent those of their affiliated organizations, or those of the publisher, the editors and the reviewers. Any product that may be evaluated in this article, or claim that may be made by its manufacturer, is not guaranteed or endorsed by the publisher.
